# Rapid Isolation and Flow Cytometry Analysis of Murine Intestinal Immune Cells After Chemically Induced Colitis

**DOI:** 10.21769/BioProtoc.5417

**Published:** 2025-08-20

**Authors:** Ashish K. Singh, Alfonso Blanco, Ray Sinnott, Ulla G. Knaus

**Affiliations:** 1 Conway Institute, School of Medicine, University College Dublin, Dublin, Ireland; 2 Flow Cytometry Core, Conway Institute, University College Dublin, Dublin, Ireland; 3 Syntec International, Dublin, Ireland

**Keywords:** Neutrophils, Immune cells, Colitis, Cell viability, Flow cytometry

## Abstract

Chemically induced murine colitis models are widely used to understand intestinal homeostasis and inflammatory responses during acute and chronic gut inflammation, such as inflammatory bowel disease (IBD). Resident populations of immune cells, together with those recruited during an inflammatory response, maintain intestinal immunity by mounting an effective immune response to enteropathogenic microbes while at the same time maintaining tolerance against commensals. To better understand the disease mechanism, studying different immune cell populations and their dynamic changes during infection and inflammation is essential. However, isolating healthy and viable immune populations, particularly hyperactivated neutrophils and macrophages from the inflamed gut (i.e., active disease site), is challenging as tissues are usually subjected to rigorous enzymatic digestion for an extended period. Here, we describe a method that uses a cell dissociator (Medimachine II from Syntec International) to separate intestinal tissue after short enzymatic digestion to obtain a single-cell suspension. This technique facilitates the isolation of immune cells from mouse intestinal tissues in high quantity and with superior viability in a very short time frame. This protocol delivers 80%–90% cell viability, which is 1.5 to 2-fold higher than conventional methods of isolating cells from inflamed mouse colons. The composition, phenotype, activation state, and gene expression profile of cells isolated using this protocol can be assessed by using multiple methods, including, but not limited to, flow cytometry, quantitative PCR, immunoblotting, mass spectrometry, single-cell RNA sequencing, and functional readouts such as reactive oxygen species (ROS) production.

Key features

• Infiltrating immune cells are key drivers of intestinal inflammation.

• Isolating viable cells from the inflamed colon is slow and may alter cell activation and expression profiles.

• This protocol enables faster isolation with improved cell viability.

• Isolated cells can be further purified using magnetic beads or flow cytometry for downstream analysis.

## Graphical overview


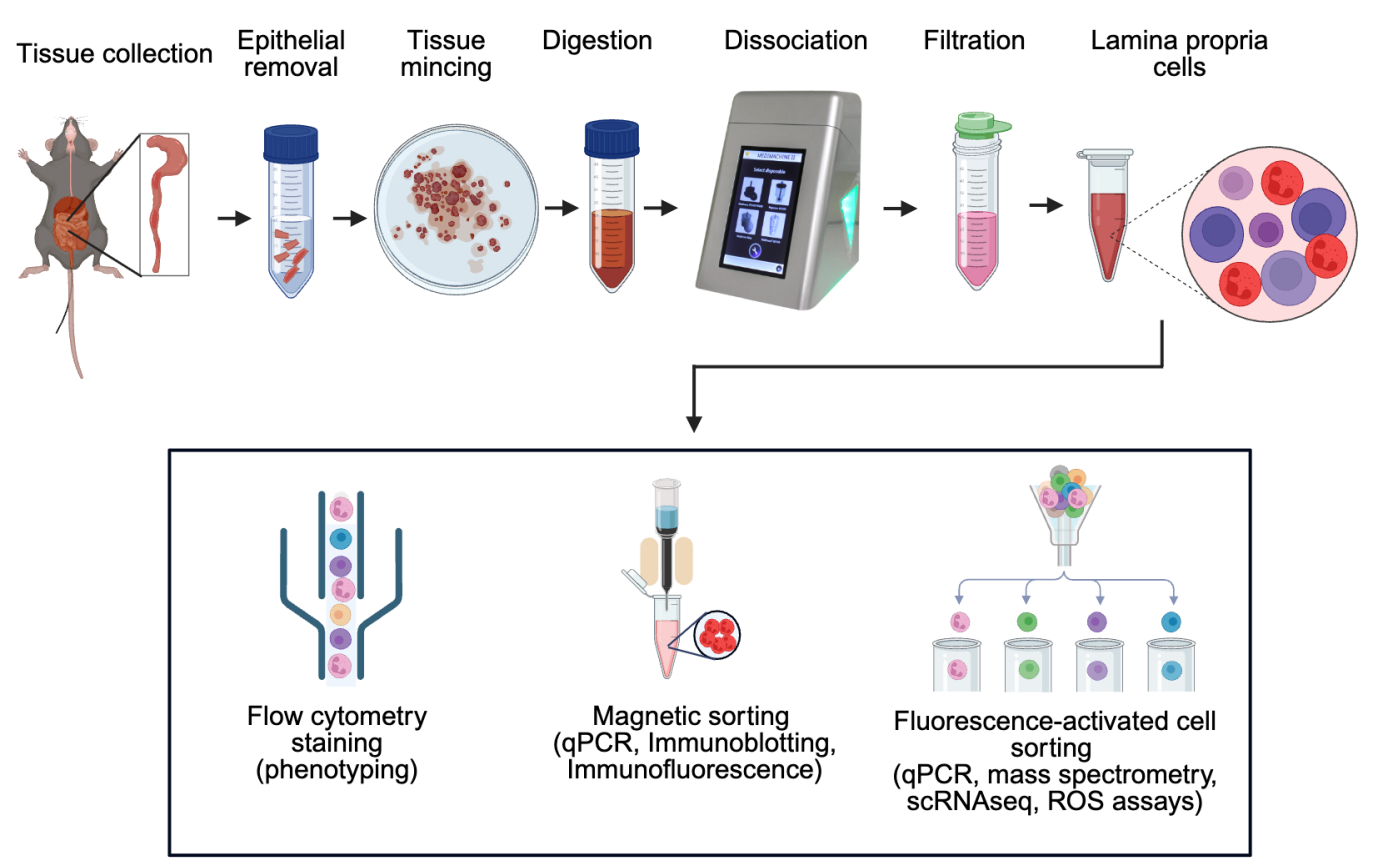


**Workflow for efficient isolation of murine intestinal immune cells via combined enzymatic and mechanical dissociation**

## Background

An intricate network of various immune cells and secreted cytokines and chemokines functions in cooperative ways to protect the host from adverse environmental conditions and microbial infections, to maintain or restore homeostasis [1]. The gastrointestinal (GI) tract is constantly exposed to commensal, opportunistic, and pathogenic microbes, creating perturbations that can lead to life-threatening infections and chronic inflammation. The host defense system in the GI tract is comprised of a single layer of epithelium fortified with adjoining cells and lymphoid tissues that operate efficiently to maintain barrier homeostasis. This barrier strikes a balance of remaining tolerant to commensal microbes and dietary antigens while preserving the capacity to mount an inflammatory response against pathogens.

Inflammatory bowel disease (IBD), a general term for chronic inflammatory conditions of the GI tract, is comprised of two main clinical entities: Crohn’s disease and ulcerative colitis. A broadly compromised or dysregulated immune system due to environmental changes, genetic background, or a qualitative and quantitative abnormal gut microbiota is a hallmark of IBD pathogenesis [2]. Despite several decades of research, our understanding of onset, recovery, and recurrent flares in IBD remains tenuous. Recruitment and activation of immune cells at inductive sites [Peyer’s patches (PP), isolated lymphoid follicles (IELs), and mesenteric lymph nodes (MLNs)] and initiation of effector functions in the lamina propria and epithelium is a sequential and complex process. In chronic disease conditions, constant activity of immune cells (i.e., ROS generation, chemokine, and cytokine secretion) in conjunction with architectural damage of tissues further induces immune cell recruitment, hence forming a perpetual destructive loop [3]. To fully comprehend the spatiotemporal dynamics of transmigrated cells and their crosstalk with other cell types, efficient isolation and characterization of distinct immune cell populations is essential. A relatively simple and rapid protocol for isolating immune cells from intestinal tissue will be highly beneficial, as intestinal immunity is at the center stage in IBD, colorectal cancer (CRC), or host–microbe interactions. Isolating healthy and viable cells from the inflamed gut, including hyperactivated neutrophils and macrophages in early acute inflammation or various T lymphocytes at later stages of the disease, is challenging but crucial for immunophenotyping and functional assays of selected cell populations. The majority of the existing protocols describe isolation of cells from normal (non-inflamed) tissues during homeostasis [4–6]; however, those that isolate from inflamed tissues have an extended digestion period and often lack viability data [7–10].

This protocol describes a straightforward method for rapid isolation of highly viable immune cells from murine intestinal tissues, such as colonic and ileal lamina propria, PP, and MLN, using Medimachine II (Syntec International, Ireland). This protocol has also been used to isolate viable cells from the murine spleen and can easily be adapted to phenotype diverse cell populations from human tissues. Here, we used chemically induced models of acute colitis in mice, such as 2,4,6-trinitrobenzene sulfonic acid (TNBS) and dextran sodium sulfate (DSS) treatment, for isolating cells from colonic lamina propria at the peak of disease.

## Materials and reagents

**Biological materials**

1. C57BL/6J mice (The Jackson Laboratory, USA)

**Reagents**

1. 70% industrial methylated spirit (70% IMS)

2. Anti-mouse CD45, Brilliant Violet 510 antibody (BioLegend, clone 30F11, catalog number: 103137)

3. Anti-mouse CD11b, APC (BioLegend, clone M1/70, catalog number: 101211)

4. Anti-mouse Ly6G, PE antibody (BioLegend, clone 1A8, catalog number: 127607)

5. Collagenase D (Sigma-Aldrich, catalog number: 11088866001), stock 500 mg/10 mL HBSS

6. Dispase (Corning, catalog number: 354235), stock 5,000 U/100 mL HBSS

7. DNase I (Sigma-Aldrich, catalog number: 10104159001), stock 100 mg/10 mL HBSS

8. DPBS, calcium and magnesium-free (Thermo Fisher Scientific, catalog number: 14190144)

9. EDTA, 500 mM in H_2_O, sterile filtered (Sigma-Aldrich, catalog number: E4884-500G)

10. FBS, heat-inactivated (Sigma-Aldrich, catalog number: F7524-500ML)

11. HBSS, calcium and magnesium-free (Thermo Fisher Scientific, catalog number: 14175095)

12. HEPES solution (Sigma-Aldrich, catalog number: H0887-100ML), 1 M

13. Liberase^TM^ (Sigma-Aldrich, catalog number: 5401119001), stock 5 mg/2 mL HBSS

14. Non-essential amino acids (Sigma-Aldrich, catalog number: M7145-100ML), 100×

15. Sodium azide (NaN_3_), 100 mM solution in sterile water (Sigma-Aldrich, catalog number: S2002-25G)

16. Penicillin-streptomycin (Thermo Fisher Scientific, catalog number: 15140122), 10,000 U/mL

17. Red blood cell (RBC) lysis buffer (Thermo Fisher Scientific, catalog number: 00-4333-57)

18. RPMI-1640 with L-glutamate, without phenol red (Gibco, catalog number: 11835-063)

19. Sodium pyruvate (Sigma-Aldrich, catalog number: S8636-100ML), 100 mM

20. Trypan Blue solution (Sigma-Aldrich, catalog number: T8154-20 ML)

21. ViaKrome 808 Fixable Viability Dye (Beckman Coulter, catalog number: C36628)

22. Fixable Viability Dye eFluor^TM^ 780 (Thermo Fisher Scientific, catalog number: 65-0865-14)

23. Anti-Mouse CD16/CD32 (Mouse BD Fc Block^TM^) (BD Biosciences, catalog number: 553142)

24. eBioscience^TM^ intracellular (IC) fixation buffer (Thermo Fisher, catalog number: 00-8222-49)

**Solutions**

1. Epithelial removal solution (see Recipes)

2. Washing solution (see Recipes)

3. 2× Digestion solution (see Recipes)

4. cRPMI (see Recipes)

5. Digestion solution (see Recipes)

6. Flow cytometry staining buffer/FACS buffer (see Recipes)

**Recipes**

**1. Epithelial removal solution (500 mL)**

Store at 4 °C for 2–3 weeks. Prewarm in the water bath at 37 °C before use.

**Table:** 

Components	Volume required	Final concentration
1 M HEPES solution	5 mL	10 mM
500 mM EDTA	5 mL	5 mM
Penicillin-Streptomycin	1 mL	50 U/mL
HBSS	489 mL	--

**2. Washing solution (500 mL)**

Store at 4 °C for 2–3 weeks.

**Table:** 

Components	Volume required	Final concentration
1 M HEPES	5 mL	10 mM
Penicillin-Streptomycin	1 mL	50 U/mL
HBSS	494 mL	--

**3. 2× Digestion solution (250 mL)**

Store at 4 °C for 2–3 weeks.

**Table:** 

Components	Volume required	Final concentration
100× non-essential amino acids	5 mL	1×
100 mM sodium pyruvate	5 mL	2 mM
1 M HEPES	5 mL	20 mM
FBS (heat-inactivated)	12.5 mL	10%
Penicillin-Streptomycin	1 mL	100 U/mL
RPMI-1640	221.5 mL	--

**4. cRPMI (600 mL)**

Store at 4 °C for 2–3 weeks.

**Table:** 

Components	Volume required	Final concentration
100× non-essential amino acids	6 mL	1×
100 mM sodium pyruvate	6 mL	1 mM
1 M HEPES	6 mL	10 mM
FBS (heat-inactivated)	60 mL	10%
Penicillin-Streptomycin	1.2 mL	50 U/mL
RPMI-1640	520.8 mL	--

**5. Digestion solution (10 mL)**

To be made fresh. Keep on ice until use. Prewarm in the water bath at 37 °C before use

**Table:** 

Components	Volume required	Final concentration
Dispase	2.5 mL	12.5 U/mL
Collagenase D	50 μL	250 μg/mL
Liberase^TM^	150 μL	37.5 μg/mL
DNase I	200 μL	200 μg/ mL
2× Digestion solution (Recipe 3)	5 mL	1×
RPMI-1640	2.1 mL	--

**6. Flow cytometry staining buffer/FACS buffer (100 mL)**

**Table:** 

Components	Volume required	Final concentration
FBS (heat-inactivated)	2 mL	2%
EDTA (500 mM)	0.4 mL	2 mM
NaN_3_ (100 mM)	2 mL	2 mM
DPBS	95.6 mL	--

**Laboratory supplies**

1. 1 and 10 mL syringes (sterile)

2. 100, 70, and 40 μm cell strainers (sterile)

3. 15 and 50 mL tubes (sterile, without skirts)

4. 6-well sterile tissue culture plates (Fisher Scientific, catalog number: 10578911)

## Equipment

1. Flow cytometer (Beckman Coulter, model: CytoFLEX LX or any other compatible cytometer)

2. Light microscope

3. Medimachine II**^®^** (Syntec International)

4. Swing-bucket Eppendorf^®^ cooling centrifuge

5. Tissue culture incubator set to 37 °C

6. Water bath set to 37 °C

7. Vortex mixer

8. Hemacytometer (Sigma-Aldrich, catalog number: Z359629-1EA)

9. Mouse dissecting tools

10. Medicons and Medicons Max (Syntec International, catalog number: 79500 S)

## Procedure

1. Euthanize C57BL/6 mice either on day 8 of oral DSS treatment or on day 2 of intrarectal TNBS instillation.

2. Spray 70% IMS on the mouse abdomen and dissect out the small or large intestine. Remove the surrounding connective tissue quickly and keep the intestinal tissue in 2 mL of ice-cold cRPMI in a 6-well plate with a lid. Move to a tissue culture room and use a biosafety cabinet category 2.

3. Cut the intestine open longitudinally to wash out feces with room-temperature (RT) washing solution. Remove excessive liquid by using blotting paper (or Kim wipes), cut tissues into approximately 1 cm pieces, and immerse them in prewarmed epithelial removal solution (30 mL/colon in a conical tube). Place horizontally in the incubator on a shaker at 37 °C and 200 rpm for 10 min.

*Notes:*

*1. The duration of the incubation should be optimized for the selected tissue. Examples are 15 min for untreated mouse ileum and 5–10 min for inflamed and damaged colon (after chemical colitis treatment with DSS or TNBS)*.

*2. If working with Peyer’s patches or spleen, skip steps 2 and 3 and directly proceed to step 5. No digestion is required for MLNs (as cells are inside the sac); hence, proceed directly to step 6 (i.e., by placing MLNs directly in Medicons).*

4. Collect tissues and wash with 30 mL of washing solution (RT) to remove EDTA. Collect the tissue in a sterile Petri dish for the next step.

5. Cut the intestinal tissues into smaller pieces on a sterile plastic Petri dish using a sterile scalpel. Mix minced tissue with 5 mL of digestion solution (freshly prepared within 30 min and prewarmed at 37 °C in the water bath) in 50 mL tubes. Place tubes vertically in the tissue culture incubator (37 °C, no shaking) for 10 min. Peyer’s patches can be directly placed in the 1–2 mL digestion solution without any mincing.

*Note: For intestinal lamina propria cell isolation, use Medicon MAX with a 16 mL volume capacity. For other tissues, such as Peyer’s patches or smaller organs, use Medicons with 1.6 mL capacity (up to five Peyer’s patches and all MLNs per one Medicon).*

6. Vortex the tubes at maximum speed for 5–10 s and collect the supernatant (containing some single cells) in a fresh 50 mL collecting tube using a 100 μm cell strainer. Move the tissue from the cell strainer into Medicons MAX by carefully placing them on the sieve using sterile tweezers. Add 15–16 mL of cRPMI (8–10 °C) through the collecting port using a syringe. Place the Medicon MAX into the corresponding slot of the Medimachine II and dissociate cells for 10 s at medium speed (10,000 rpm). Collect the liquid from the Medicons using a sterile syringe and combine it into the collecting tube above by using a 100 μm cell strainer. Add cRPMI (8–10 °C) to a final volume of 45 mL and keep the tube on ice until the next step.

*Note: Move Peyer’s patches directly into the Medicons from the digestion solution without vortexing, add 1.6 mL of cRPMI (8–10 °C), and proceed for cell dissociation as described above in step 5. As mentioned, MLNs do not need digestion; thus, move all MLNs directly into the Medicons. Collect cells through a 70 μm cell strainer into 15 mL tubes and add cRPMI to a final volume of 10 mL.*

7. Centrifuge the collecting tubes containing cells at 450× *g* for 15 min at 10 °C. Discard the supernatant and proceed with the remaining cells to red blood cell lysis using RBC lysis buffer if required (see note below). Resuspend the pellet carefully with 35 mL of cRPMI (8–10 °C) and filter through a 40 μm cell strainer into a new tube.

*Note: If the pellet has reddish coloration, it usually requires RBC lysis. This is often observed in inflamed colonic tissues. Gently resuspend the cell pellet in 500 μL of ice-cold RBC lysis buffer and allow lysis for 3–4 min at room temperature. Add 10 mL of cRPMI (8–10 °C) and centrifuge at 450× g for 15 min at 10 °C. Wash once more with 10 mL of cRPMI (8–10 °C) before proceeding.*

8. Centrifuge tube at 450× *g* for 15 min at 10 °C, discard the supernatant, and resuspend the pellet in the desired volume of cRPMI (for example, 2 mL for colonic or ileal lamina propria and 1 mL for Peyer’s patches or MLNs).

9. Viability can be immediately assessed by the Trypan Blue exclusion test. Mix cell suspension and Trypan Blue in a 1:1 ratio, load the mixture into a hematocytometer, and count cells under a light microscope. Viable cells are impermeable to Trypan Blue, whereas the dye permeates into dead cells, which appear blue under a light microscope. For flow cytometry analysis, cells can be stained with chosen dyes and antibodies, as demonstrated in this study. A brief protocol for staining for flow cytometry is as follows:

a. Resuspend 10^6^ cells in 100 μL of 10 mM HEPES buffer containing live dead dye (Fixable Viability Dye) in the concentration suggested by the manufacturer. Incubate for 15 min in the fridge, followed by washing with 10 mM HEPES and centrifuging at 450× *g* for 6 min at 10 °C.

b. Prepare a 1:100 dilution of Fc-blocking solution in FACS buffer (anti-mouse CD16/CD32 antibodies cocktail). Add 10 μL/tube of Fc-blocking solution and mix well. Incubate at room temperature for 5 min and add antibodies directed to cell surface proteins in suitable concentrations (CD45, 1:100; Ly6G, 1:50; CD11b, 1:100). Add flow cytometry staining buffer (Recipe 6) up to 100 μL. Incubate in the dark at 4 °C for 20 min, followed by washing twice with FACS buffer and centrifuging at 450× *g* for 6 min at 10 °C.

c. To fix and store stained cells for later analysis, dilute intracellular (IC) fixation buffer with FACS buffer in a 1:1 ratio. Resuspend the cell pellet obtained above with 100 μL of diluted IC fixation buffer. Keep in the dark at 4 °C until analysis.

**Figure 1.**
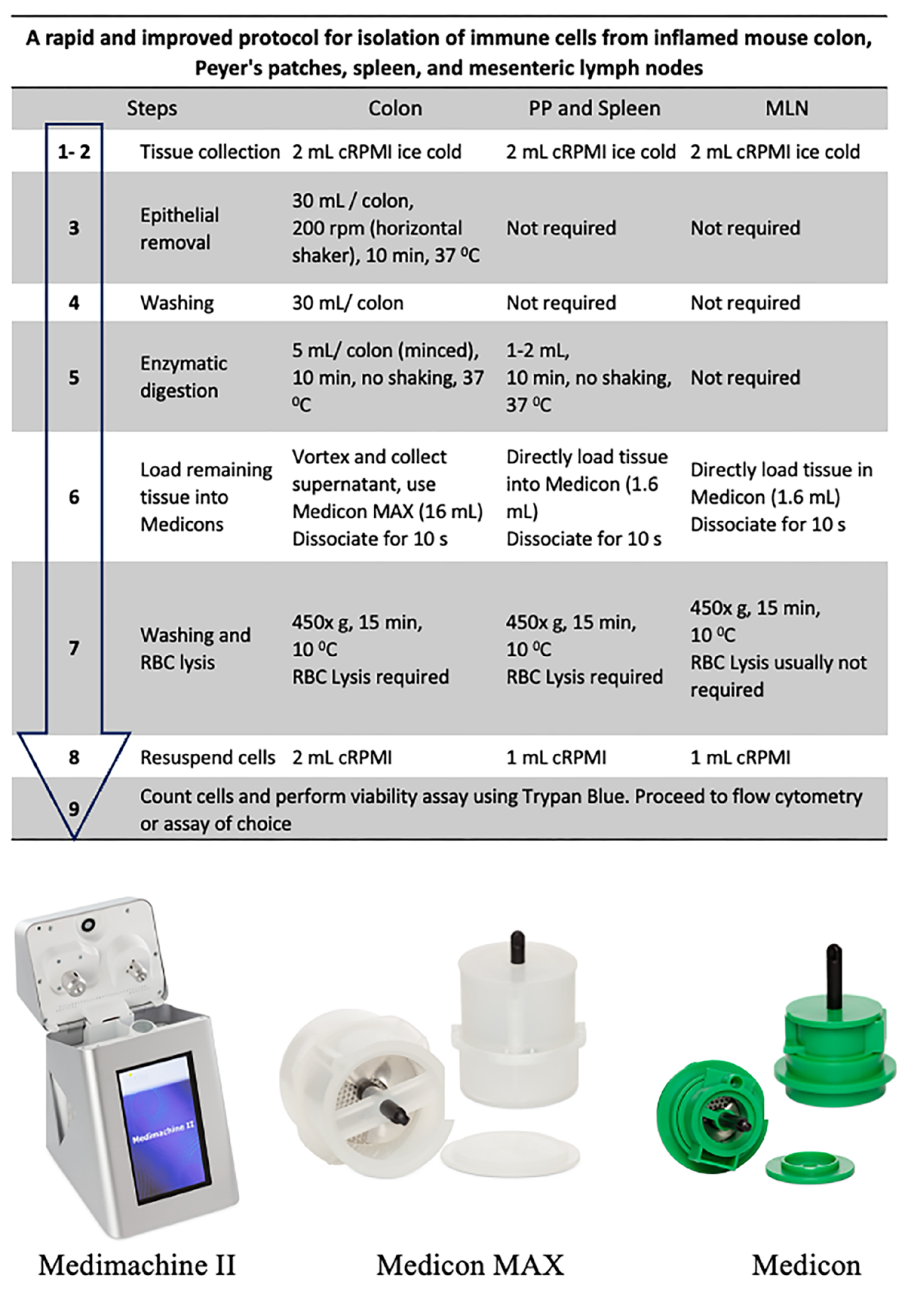
Flow diagram of the isolation procedure (top) and representative images of Medimachine II and Medicons (bottom)

A summary of distinguishing steps and different volumes required for a variety of tissues (colon, Peyer’s patches, spleen, and MLNs) is shown in Figure 1.

## Data analysis

**Figure 2.**
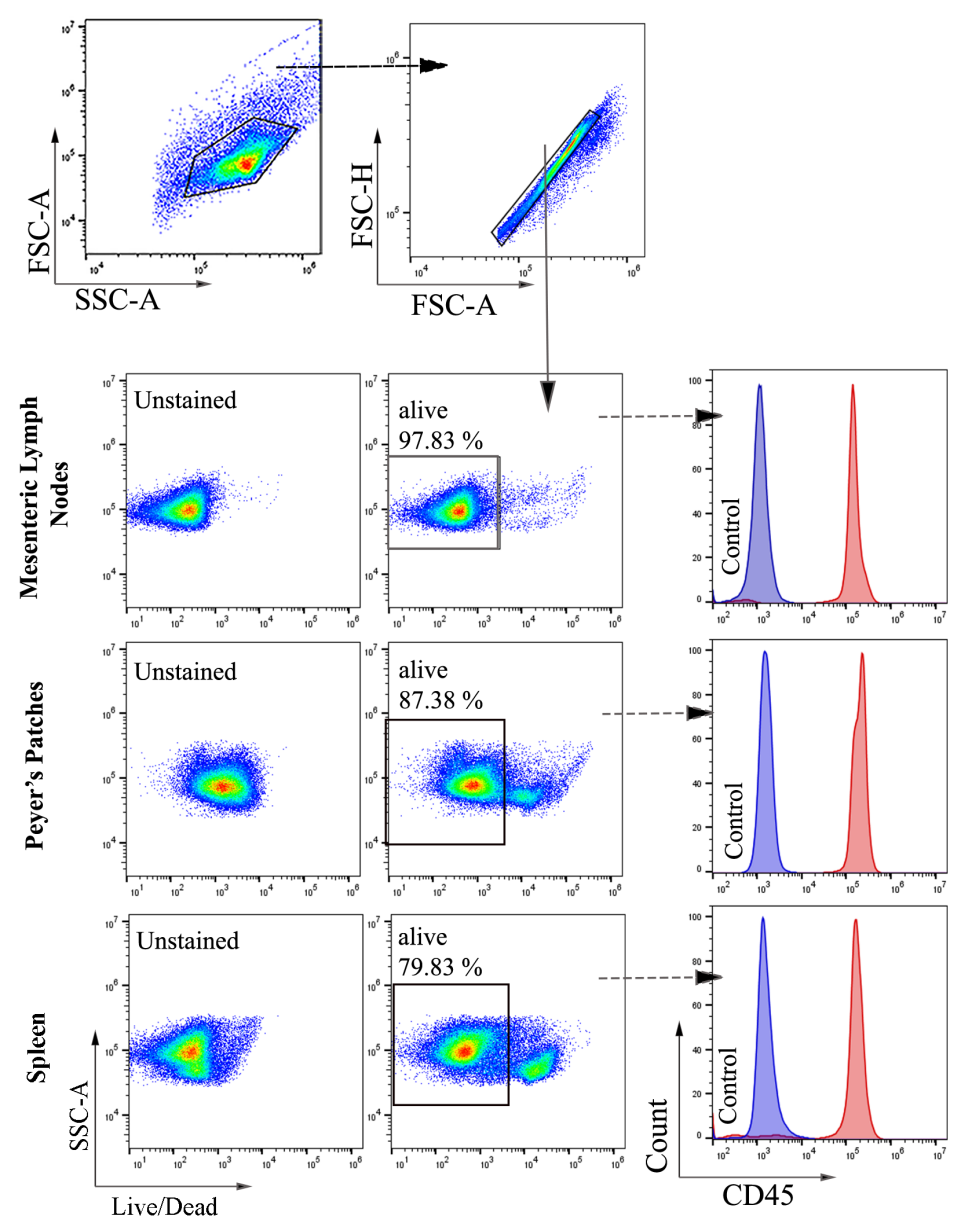
Representative images of flow cytometry analysis and gating strategy of cells extracted from mesenteric lymph nodes (MLNs), Peyer’s patches (PP), and spleen (A) of untreated mice. Total cells were selected based on forward and side scatter, followed by selecting singlets. From singlets, the Fixable Viable Dye eFluor^TM^ 780 negative population (alive) was selected for further gating of CD45-positive cells. Filled blue and red histograms represent unstained and CD45 Brilliant Violet 510-stained cells, respectively (n = 6).

**Figure 3.**
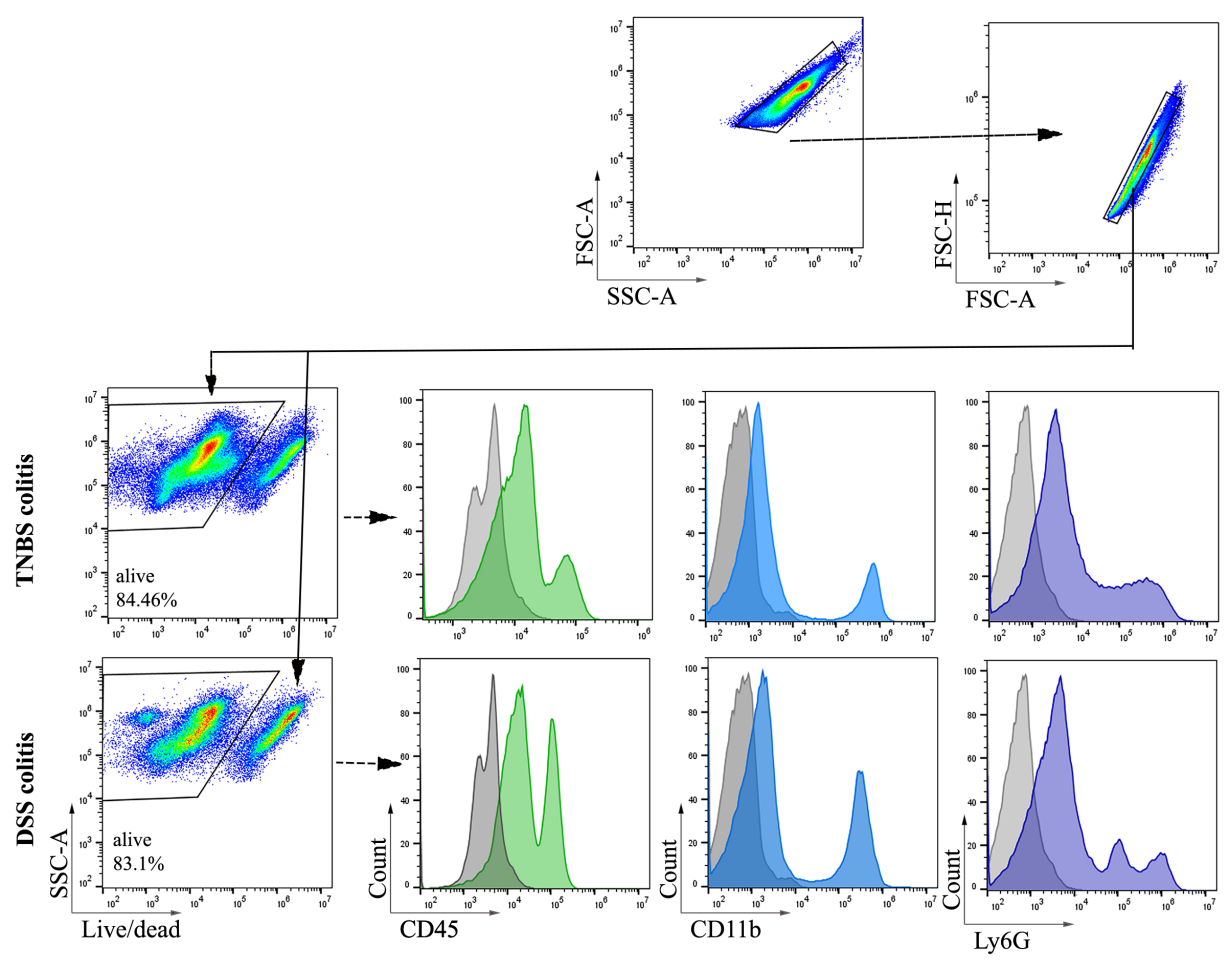
Representative images of flow cytometry analysis and gating strategy of cells extracted from C57BL/6 mice colonic lamina propria after dextran sodium sulfate (DSS)- and trinitrobenzene sulfonic acid (TNBS)-induced colitis. Colonic tissues were harvested after 48 h (day 2) of 2.5% TNBS enema. For DSS-induced colitis, colonic tissues were collected on day 8 (6 days of oral 3% DSS treatment followed by water). Total cells were selected based on forward and side scatter, followed by selecting singlets. ViaKrome 808 Fixable Viability Dye negative populations (alive) derived from singlets were selected for gating of CD45-, CD11b-, and Ly6G-positive populations. A filled grey histogram represents unstained (FMO) cells. Filled green, blue, and purple histograms represent CD45-, CD11b-, and Ly6G-positive populations (n = 6).

**Figure 4.**
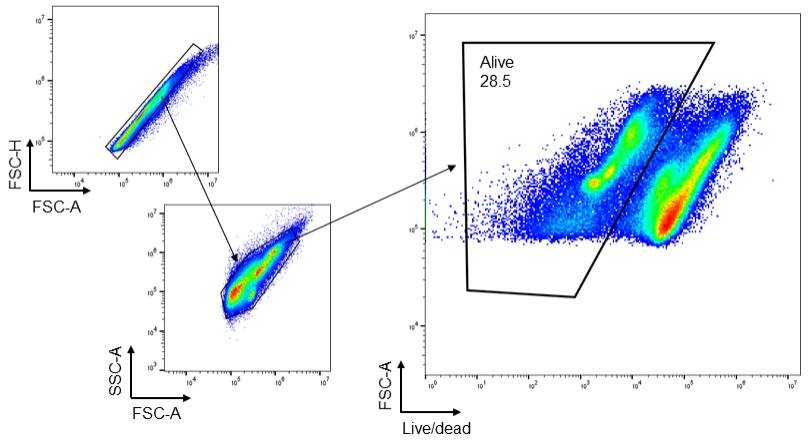
Representative images of flow cytometry analysis of cells extracted from trinitrobenzene sulfonic acid (TNBS)-induced colitis mouse colon using the conventional method. Colonic tissues were harvested after 48 h (day 2) of 2.5% TNBS enema. After selecting singlets, total cells were gated, followed by gating live/dead cells. Fixable Viability Dye eFluor^TM^ 780 was used to distinguish alive (negative staining) and dead (positive staining) cells (n = 3).

1. In this study, colon samples were stained with ViaKrome 808 Fixable Viability Dye, and other samples (MLN, PP, and spleen) were stained with Fixable Viability Dye eFluor^TM^ 780. CD45 was used to detect all leukocytes, as shown in Figures 2 and 3. CD11b- (macrophages and neutrophils) and Ly6G (neutrophils)-positive cells from colonic tissue (DSS- and TNBS-induced colitis) are shown in Figure 3. Consistent cell viability of 80%–90% was observed in cells extracted from colonic lamina propria after colitis. Distinct CD45-, CD11b-, and Ly6G-positive populations are shown in histograms.

2. Several widely used methods for isolating immune cells from intestinal tissues require the use of enzymes such as collagenases, dispases, and DNase I [5,11]. These methods include digestion of intestinal tissues with these enzymes for 30–45 min at 37 °C with vigorous shaking. The prolonged time required for cell isolation and the mechanical stress of shaking negatively affect cell viability (Figure 4) and, most importantly, can alter the activation state of innate immune cells (especially neutrophils and macrophages). The incubation time is the most prominent constraint. By reducing enzyme concentration and exposure time, together with an efficient extraction technique, we developed a fast and reliable method for the extraction of viable immune cell populations from the mouse intestine. In preliminary experiments, different incubation times and enzyme concentrations were used, and we found the proposed duration and concentration optimal in our conditions. When establishing this protocol for the first time in the laboratory, crucial steps such as epithelium removal, tissue digestion, and dissociation using Medimachine II should be optimized for the specific tissue. For example, when using a control (non-inflamed) colon, the duration of epithelium removal (step 2) should be increased and optimized to achieve complete removal of the epithelium. Likewise, if colonic tissue is expected to be severely damaged, and massive loss of epithelium has been observed, it is recommended that the duration of the epithelial removal step (step 2) is decreased to prevent loss of lamina propria cells.

## Validation of protocol

This protocol or parts of it have been used and validated in the following research article:

• Singh et al. [12]. De novo DUOX2 expression in neutrophil subsets shapes the pathogenesis of intestinal disease. *Proc. Natl. Acad. Sci. U.S.A.* (Figures 1, 2, 4, and 5)

## General notes and troubleshooting

1. If this protocol is to be used for downstream analysis such as qPCR, RNAseq, or functional assays (e.g., ROS assays, cytokine production, etc.), euthanize mice in sterile conditions in a BSL2 cabinet.

2. Remove all fat tissue attached to the intestine after excision before proceeding to the next step.

3. If the aim is to count the total number of infiltrated immune cells (e.g., neutrophils), collect the washing solution suspension (step 3), filter it through a 40 μm cell strainer, and combine it with the cells isolated in step 7.

4. If the lamina propria cell pellet obtained in step 7 shows visible mucus (whitish coloration), especially in colonic and ilial tissues, it is recommended to dissolve the mucus with dithiothreitol (DTT) before proceeding to RBC lysis. Use sterile 10 mM DTT to resuspend the pellet and incubate for 5 min at RT, followed by washing with 35 mL of cRPMI.

5. If lamina propria cell suspension is to be used for cell sorting, it is advised that cells be resuspended and sorted in the recommended compatible solution. For example, bovine serum albumin (BSA) and FBS-free media were used for proteomics analysis, and 0.4% BSA in RPMI-1640 was used for RNAseq analysis of neutrophils in the validated study [12].
